# A systematic review of the association between parent‐child communication and adolescent mental health

**DOI:** 10.1002/jcv2.12205

**Published:** 2023-11-04

**Authors:** Holger Zapf, Johannes Boettcher, Yngvild Haukeland, Stian Orm, Sarah Coslar, Krister Fjermestad

**Affiliations:** ^1^ Department of Child and Adolescent Psychiatry, Psychotherapy and Psychosomatics University Medical Center Hamburg‐Eppendorf Hamburg Germany; ^2^ Adults for Children Foundation Oslo Norway; ^3^ Division Mental Health Care Innlandet Hospital Trust Oslo Norway; ^4^ Frambu Resource Centre for Rare Disorders Oslo Norway; ^5^ Faculty of Behavioral and Social Sciences University of Groningen Groningen The Netherlands; ^6^ Department of Psychology University of Oslo Oslo Norway

**Keywords:** child mental health, parent‐adolescent communication, parent‐adolescent relationship, review

## Abstract

**Background:**

This systematic review addresses how adolescent‐rated parent‐child communication (PCC) quality is related to adolescent mental health.

**Methods:**

We performed a systematic literature search in Medline and APA PsycInfo, including peer‐reviewed quantitative studies examining associations between adolescent‐rated dyadic PCC quality and general as well as specific measures of adolescent mental health. Qualitative and case studies were excluded, as were studies reporting only parent‐rated communication quality or instruments assessing other constructs than dyadic PCC. We screened 5314 articles, of which 37 were included in the review. We assessed study quality with the Mixed Methods Appraisal Tool.

**Results:**

We synthesized the findings in a table and narratively, reporting the main outcomes organized according to mental health constructs. The included studies were mainly cross‐sectional. The results showed that adolescent‐rated PCC quality is negatively associated with mental health constructs, demonstrating small to large effects across different mental health constructs and populations. The associations were found for general mental health and specific domains, including depression, anxiety, psychosis, suicidal ideation, post‐traumatic stress symptoms, and addictive internet use/gaming.

**Conclusions:**

The findings demonstrate that PCC is a relevant variable to consider in assessing adolescent mental health and preventive efforts. Limitations include the exclusive focus on adolescent‐reported PCC questionnaires since parent‐ and observer‐rated instruments may lead to different results. Also, PCC is related to other constructs, such as dyadic relationships, that were not included in this review. We conclude that PCC is a relevant variable to consider in mental health research. Our findings suggest that PCC may be considered in mental health practice, both in terms of assessing its quality and potentially by tailoring interventions to enhance PCC. These may represent a mean to promote adolescent mental health.

**Key points**

- This systematic review is the first to give a comprehensive overview of the relationship between different areas of adolescent mental health and the quality of parent‐child communication.
- Across the included studies, parent‐child communication quality is linked to adolescent mental health with mostly small to medium associations.
- Results suggest that parent‐child communication is of therapeutic relevance especially in adolescents with depression, post‐traumatic stress disorder and psychosis.
- More interventional research is needed to improve the evidence base.

## INTRODUCTION

Interpersonal dyadic communication comprises verbal, para‐verbal, and non‐verbal two‐way interactions that can express feelings, thoughts, values, and needs (Guerrero et al., 2017). Within a family, a high quality of dyadic communication means that the dyad will be able to better deal with problems and developmental demands (Olson et al., 2019). Typical operationalizations of the dyadic communication concept include the degree of ease with which one self‐discloses to the other person, the degree of trust one places in the statements of the other, or the level of conflicts that show up in communication, for example, insults (Barnes & Olson, 1985). In terms of dyads within families, research has especially focused on couple and parent‐child dyads.

In complex ways, the quality of interpersonal communication is associated with mental health, defined as a state of internal equilibrium, entailing the ability to cope with adverse life events, express and regulate emotions, empathize with other individuals, and perform social roles (Galderisi et al., 2017; Kiesler, 1992). This broad definition can be considered as two ends of a continuum; one focusing on positive aspects promoting equilibrium, such as empathizing with others and being able to perform social roles (i.e., social and emotional competencies), and one focusing on negative aspects or symptoms of disequilibrium, such as a lack of ability to cope with stressful or adverse events, difficulties with regulating negative emotions of sadness, aggression, and anxiety, and inability to adapt to social norms. The presence of disequilibrium, or negative aspects, can be considered a mental health problem and is the focus of the current study. It is assumed that problems in communication are associated with specific mental health problems such as depression and psychosis, as well as general symptom burden in the field of mental health problems without a specific diagnosis (Watzlawick et al., 2011).

To the best of our knowledge, three different models for the relationship between mental health and communication have been proposed and discussed in the literature. The first model assumes a bidirectional relationship between communication quality and mental health. For specific disorders such as depression or eating disorders, some evidence for reciprocal processes between mental health symptoms and communication has been demonstrated in reviews and observational studies (Chiariello & Orvaschel, 1995; Prescott & Le Poire, 2002). That is, disorder symptoms increase the likelihood of communication difficulties which, in turn, increase the symptom burden. In this reciprocal process model, no starting point is specified. Symptom reduction or improvement of communication may lead to a virtuous cycle, just as symptom increase or worsening of communication may lead to a vicious cycle. Further clarification of these relationships is still pending. It is not yet clear whether reciprocal dynamics are limited to certain developmental periods or specific diagnoses. In a second model, communication quality is considered a direct potential risk/protective factor for mental health. Empirical studies have shown that mental health difficulties are associated with poorer communication quality in families, couples, and in the workplace, while higher communication quality is considered a resilience/protective factor (Elgar et al., 2013; Niedhammer et al., 2015; O’Shea et al., 2014; Segrin, 2006; Sher & Baucom, 1993). Finally, in a third model, communication quality is considered a mediator proposed to explain the association between risk or protective factors and mental health (Riesch et al., 2006). For example, the association between interparental conflict and depressive symptoms in adolescent children has been demonstrated to be mediated by parent‐child communication (PCC) (Ying et al., 2018). In a similar vein, it has been demonstrated that high communication quality mediates the relationship between cohesive‐flexible family functioning and depression and anxiety (Berryhill et al., 2018). In the present review, we considered evidence for the aforementioned models and general evidence for the association between PCC quality and adolescent mental health.

Parent‐child communication is life's first enduring dyadic long‐term interaction. This interaction represents a formative experience (Lambert & Cashwell, 2004). Parents act as models for communication and shape children's communication behavior and their ability to express needs and emotions, and the quality of early PCC also influences attachment and mentalization (Luyten et al., 2020; Segrin, 2006). Across the developmental span, the quality of PCC also affects several other individual traits linked to mental health, such as the child's self‐esteem, school performance, and conflict management skills (Branje, 2008; Kernis et al., 2000; Noller & Feeney, 2004). Like other forms of dyadic communication, PCC is not shaped unidirectionally by the parent; rather, members of the dyad reciprocally influence each other (Segrin & Flora, 2011).

During adolescence, communication needs to be renegotiated in a process that is often accompanied by conflict (Keijsers & Poulin, 2013; Laursen & Collins, 2004). Thus, maintaining high‐quality PCC becomes more challenging in this period of life, which is marked by a higher vulnerability of developing a mental health disorder relative to other developmental stages (Klasen et al., 2016). Nevertheless, studies suggest that PCC quality and adolescent mental health are still closely related, even though the importance of the parent‐child relationship decreases relative to that of the peer group (Manczak et al., 2018; Wang et al., 2020; Wu & Chao, 2011). Accordingly, targeting PCC is a valuable aim in therapy for children and adolescents (Robin & Foster, 2002), and preventive mental health interventions targeting the improvement of PCC have been developed recently (Toombs et al., 2018; Vatne et al., 2019).

The association between PCC quality and adolescent mental health has been studied with different designs, using parent‐, child‐, or observer‐rated measures and focusing on different populations concerning general mental health or specific domains like depression, abuse, or addictive behavior (Ioffe et al., 2020; Lee et al., 2018; Reis & Heppner, 1993; Young & Childs, 1994). Although the importance of PCC for adolescent mental health seems to be well‐established and studied in relation to specific disorders and general mental health, there has been little effort to synthesize the findings systematically. Concerning observer‐rated measures, a systematic review of PCC and child anxiety was performed in 2016, finding evidence for the association between observer‐rated parental verbal communication and child anxiety (Percy et al., 2016). However, to our knowledge, in contrast to related constructs such as attachment (Lam et al., 2019; Wylock et al., 2021), no review has yet been conducted on the association between adolescent‐rated quality of PCC and mental health outcomes. The focus on adolescent‐rated PCC is important since data from adolescents is usually more difficult to obtain. Furthermore, the adolescents' perspective often remains underrepresented in research, even though the adolescents' perception of PCC may contain important information (Xiao et al., 2011). Systematic research is needed to understand if and when adolescent‐reported PCC is a relevant predictor for adolescent mental health in general and in terms of specific mental disorders. Such systematic knowledge will also shed light on whether the relations between adolescent‐reported PCC and different disorders show different patterns.

In the current review, we aim to synthesize the recent literature on the association of adolescent‐reported PCC quality and adolescent mental health. We examine the results across different areas of psychosocial functioning and mental health.

## METHODS

This review is based on a systematic literature search that was conducted to identify adolescent‐rated measures for PCC (Zapf et al., 2023), Prospero Registration: CRD42021255264. For the present study, we conducted an updated search and refined eligibility criteria by solely including studies that analyzed associations between PCC and adolescent mental health. We report our findings according to the Preferred Reporting Items for Systematic Reviews and Meta‐Analyses (PRISMA) guidelines (Page et al., 2021).

### Eligibility criteria

To be eligible, studies had to be original, peer‐reviewed journal articles published in English assessing the quality of dyadic communication between parents and their adolescent children and containing at least one measure of adolescent mental health linked to PCC in the analysis. The dyadic communication measure must have included adolescents' self‐reports and involved more than one item. It had to focus explicitly on the construct of communication; hence, the search word was communication. The adolescent mental health measure had to refer to general mental health or specific areas of mental health such as conduct problems or depression. The search words used to operationalize the mental health outcomes were: Functioning or well‐being or mental health or stress or psychopathol* or adjust* or internali* or externali*. To not include underpowered studies, the sample sizes had to be at least *n* = 50. Studies assessing broader concepts such as general family communication, other concepts such as attachment styles or dyadic relationships or using measures that included communication only as a subscale of a broader concept/scale, or using single questions or ad hoc measures to assess PCC were excluded. Studies on specific communication topics such as health‐related behaviors (e.g., sex, alcohol, tobacco use) were excluded. Studies using only parents as informants on communication were excluded. The age range of the study population was set to 8–21 years of age, including older children, adolescents, and emerging adults. We included studies examining PCC in general, at‐risk, and clinical populations. Regarding study design, we included all types of empirical studies (cross‐sectional, longitudinal, interventional, and validation studies). We did not include qualitative studies.

### Data sources and search strategy

The search and selection process for the original study took place between May 2021 and February 2022 and was based on the electronic databases APA PsycInfo (Ovid) and MEDLINE (Ovid). The additional search and selection process for the present study took place between January and March 2023. On 7.2.2023, an updated search for papers was conducted, resulting in the addition of three papers. The references of all selected publications were searched for additional studies. See Zapf et al. (2023) for the original search strategy.

### Study selection process

Bibliographical data were uploaded to the Rayyan web‐based system for review scorings (rayyan.ai) for masked screening. Author pairs screened titles and abstracts. Full texts retrieved after screening were checked for eligibility by the same pairs independently, again using Rayyan. Disagreements were resolved by discussion.

### Data extraction and quality assessment

Data extracted from the studies were sample description, adolescent sample age, the focus of the paper, scale names, mental health constructs, main method, and the statistical relation of PCC to mental health constructs. Multiple reports from the same study/sample were treated as a single study. Data were extracted by pairs. Since the reviewed articles comprised methodologically diverse studies, we used the Mixed Methods Appraisal Tool (MMAT; Hong et al., 2018) for quality assessment. The focus of the quality assessment was the quality of the evidence relevant to the research question of this review, that is, the associations between PCC and mental health, not the overall study quality. The threshold for complete outcome data was set to 30% for longitudinal and intervention studies, cross‐sectional studies had to report sufficient retrieval rates (>30%) and missing values (<20%). Quality assessment was conducted in masked author pairs. Disagreements were resolved by discussion. In terms of results, we considered *r* > 0.10 small, >0.30 medium, and >0.50 large associations and *d* > 0.2 small, >0.5 medium, and >0.8 large effect sizes, respectively (Cohen, 1992).

## RESULTS

**FIGURE 1:**
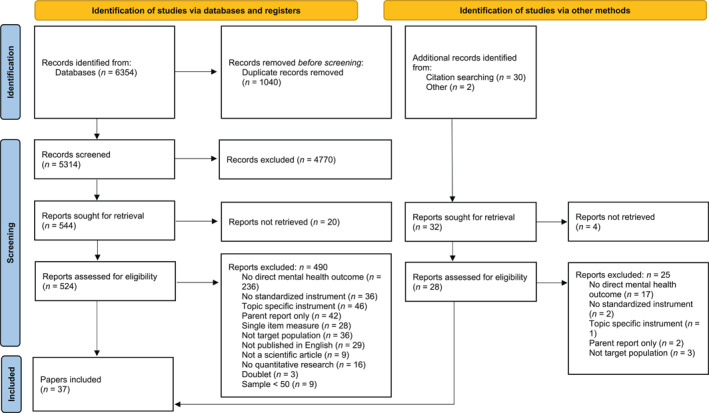
PRISMA 2020 flow diagram for new systematic reviews which included searches of databases, registers and other sources. Source: Page et al. (2021). For more information, visit: http://www.prisma‐statement.org/.

In total, we retrieved 6354 records from Medline and PsycInfo (6147 from the systematic database search from our initial study (Zapf et al., 2023) and 207 from the updated search). From these, 1040 duplicates were removed before screening. The remaining 5314 titles and abstracts were screened. Of these, 524 full‐texts were retrieved, of which 34 were included (31 from the initial study and three from the updated search). Three more papers were identified via citation searching, resulting in a total of 37 papers. See Figure 1 for the corresponding PRISMA flow chart. The 37 papers are based on 26 unique studies. Table 1 provides an overview of the included papers. In the presentation of results, we first give an overview of the type of studies we included and the types of measures, and then we present the quality assessment. Some papers focused on general mental health, whereas others focused on specific disorders. We organized the results of these studies from general to more specific mental health outcomes.

**TABLE 1:** Overview of included studies and results.

Mental health variable(s) and measure(s)	Study	Country	Sample (N, female in %)	Age (Years, M, SD, range)	PCC scale	Main findings
Addictive behavior: Internet addiction (Young's internet addiction test)	Cai et al. (2021)^CS^	China	Left‐behind (699, 45%) and non‐left‐behind children (740, 46%)	Left‐behind children (*M =* 12.4, *SD =* 1.2); non‐left‐behind children (*M =* 12.5, *SD =* 1.2)	PACS	Odds ratios: Mother PCC and internet addiction: OR = 0.99; father PCC and Internet addiction: OR = 0.94.
Addictive behavior: Internet addiction (Adolescent Pathological Internet Use Scale APIUS)	Liu et al. (2019)^CS^	China	Regular population school students (2751, 51%)	*M =* 14.9, *SD =* 1.9, range = 13–19	PACS	Correlation: PCC and pathological internet use: *r* = −0.29.
						Multiple regression: Effect of PCC on pathological internet use: *β* = −0.24, *p* < 0.001, mediated by parental responsiveness.
Addictive behavior: Internet gaming disorder (Internet Game Use‐Elicited Symptom Screen IGUESS)	Kim et al. (2018)^CS^	Korea	Regular population first‐year middle school students (402, 45%)	Not reported (first year middle school)	PACS	Correlations: Mother PCC and risk of internet gaming disorder: *r* = −0.05; father PCC and risk of internet gaming disorder: *r* = −0.38
						Multiple regression: Indirect effect of father PCC in the relationship between aggression and the risk of internet gaming disorder: *β* = 0.13.
Anxiety (SCARED^a^)	Simpson et al. (2020)^CS^	USA	Regular population high school students (1034, 55%)	*M =* 16.2, *SD =* 0.8	PACS	Correlations: Mother PCC and anxiety: *r* = −0.24 (girls) and *r* = −0.06 (boys); father PCC and anxiety: *r* = −0.15 (girls) and *r* = −0.15 (boys).
						Multiple regression: PCC mother and father predicted some aspects of coping in boys (*β* = −0.04–0.08), but were poor predictors of coping in girls.
Anxiety (Revised Children's Manifest Anxiety Scale RCMAS)	Varela et al. (2009)^CS^	USA/Mexico	Regular population school students (217, not reported)	*M =* 11.3, *SD =* 5.5, range = 9–14	PACS	Multiple regression: Better PCC with father was related to less anxiety as reported by mothers (*β* = 0.18, *p* < 0.05) in a model with other predictors, PCC alone was not a predictor of anxiety.
Depression (CES‐D^b^)	Bireda and Pillay (2018)^CS^	Ethiopia	Regular population school students (809, 47%)	*M =* 16.8, *SD =* 1.6	PACS	Correlations: Mother PCC and depression: *r* = −0.85; father PCC and depression: *r* = −0.84.
Depression (CES‐D^b^)	Chen et al. (2019)^CS^	China	Regular population junior high school students (1134, 47%)	*M =* 13.2, *SD =* 1.1, range = 11–16	PACS	Correlations: PCC and depression: *r* = −0.38. PCC and smoking behavior: *r* = −0.24.
Depression (CES‐D^b^)	Fernandez et al. (2022)^CS^	USA	Regular population hispanic adolescents (456, 53%)	*M =* 13.9, *SD =* 1.4, range 12–16	PACS	Correlation: PCC and depression: *r* = −0.38.
Depression (CES‐D^b^)	Finan et al. (2018)^L^	USA	Regular population high school students (1033, 55% at time 1)	*M* = 16.1, *SD =* 0.7 (at time 1)	PACS	Correlations: Mother PCC and depressive symptoms at time 1: *r* = −0.57; father PCC and depressive symptoms at time 1: *r* = −0.30.
						Growth curve model: Mother PCC predicted decline in depression for girls and father PCC predicted decline in depression for boys 7 years later.
Depression (CES‐D^b^)	Ohannessian and Vannucci (2020)^L^	USA	Regular population high school students (1057, 53% at time 1)	*M =* 16.2, *SD =* 0.8 (at time 1)	PACS	Correlations: Mother PCC and depression: *r* = −0.36; father PCC and depression: *r* = −0.28.
						Multiple regression: Depressive symptoms in adolescents predicted decreased PCC with mothers, but not fathers, 2 years later.
Depression (CES‐D^b^)	Rodrigues et al. (2020)^CS^	USA	Girls' summer science camp participants (95, 100%)	*M =* 15.4, *SD =* 0.1, range = 13–18	PACS	Correlations: Mother open PCC and depression: *r* = −0.44; father open PCC open and depression: *r* = −0.51.
Depression (CES‐D^b^)	Schuster et al. (2013)^CS^	USA	Regular population high school students (1145, 58%)	*M =* 16.9, range 15–19	PACS	Correlations: PCC at time 2 and depression time 1 girls: *r* = −0.28 and boys: *r* = −0.41. Cross‐sectional correlations at time 1 were exactly the same.
Depression (CES‐D^b^)	Wang et al. (2022)^CS^	China	Regular population school students (4213, 48%)	*M =* 16.4, *SD =* 0.8, range = 10–20	PACS	Correlations: Mother PCC and depression: *r* = −0.33; father PCC and depression: *r* = −0.32.
Depression (CES‐D^b^)	Ying et al. (2018)^CS^	China	Regular population, (437, 45%)	*M =* 10.9, *SD =* 0.7	Parent‐child communication questionnaire (PCCQ)	Correlations: PCC subscale open expression and depression: *r* = −0.44; subscale listening to parents: *r* = −0.43; subscale conflict resolution: *r* = −0.40; subscale mutual understanding: *r* = −0.43.
Depression (CES‐D^b^) & anxiety (SCARED^a^)	Ohannessian (2012)^L^	USA	Regular population high school students (683, 57%)	*M =* 16.1, *SD =* 0.7, range = 15–17 (at time 1)	PACS	Correlations: Mother PCC and depression: *r* = −0.22; father PCC and depression: *r* = −0.19. Mother PCC and anxiety: *r* = −0.07; father PCC and anxiety: *r* = −0.19.
						Path analysis: No direct paths between PCC and anxiety and depression in boys and anxiety in girls, direct path between mother PCC and depression in girls (*β* = −0.16, *p* < 0.001).
Depression (CES‐D^b^) & anxiety (SCARED^a^)	Pantaleao and Ohannessian (2019)^L^	USA	Regular population high school students (980, 53%)	*M =* 16.15, *SD =* 0.75 (at time 1)	PACS	Correlations: Mother PCC and depression *r* = −0.27; mother PCC and anxiety: *r* = −0.06; father PCC and depression *r* = −0.31; father PCC and anxiety *r* = −0.16
						Path analysis: Direct path between father PCC and anxiety (*β* = −0.25, *p* < 0.01) and depression (*β* = −0.19, *p* > 0.05) in boys. Direct path between mother PCC and anxiety (*β* = 0.18, *p* < 0.01) in girls.
Depression (CDI) & anxiety (SCARED^a^)	Van Dijk et al. (2014)^L^	Netherlands	Regular population high school students (323, 51.1%)	*M =* 13.3, *SD =* 0.5, range = 12–15 years	PACS	Correlations: PCC and depressive symptoms cross‐sectional: *r* = −0.37 to −0.31, longitudinal: *r* = −0.32 to −0.16. PCC and depressive symptoms cross‐sectional: *r* = −0.23 to −0.19, longitudinal: *r* = −0.20 to −0.11).
						Cross‐lagged path analysis: An indirect longitudinal path between open PCC and depression was found from time 2 to time 4, via self‐concept at time 3.
Depression & anxiety YSR^c^ subscales)	Fite et al. (2014)^L^	USA	Regular population public school students (289, 0%)	*M =* 16 at baseline	PCCS (Loeber)	Correlations: Poor PCC and depression (*r* = 0.19) and anxiety (*r* = 0.15) cross‐sectional. Poor PCC and depression (*r* = 0.14) and anxiety (*r* = 0.10) longitudinal (3 years).
						Regression analysis: No direct effect of PCC on depression and anxiety longitudinally, but interaction effect of poor PCC and reactive aggression for depression (*β* = 0.15) and anxiety (*β* = 0.18).
Depression (CES‐D^b^) & anxiety (SCARED^a^)	Ioffe et al. (2020)^L^	USA	Regular population middle school students (400, 54%)	*M =* 12.5, *SD =* 1.0, range 11–14	PACS (open communication subscale)	Correlations: Open PCC with mother and depression cross‐sectional: *r* = −0.32, longitudinal: *r* = −0.30, father PCC and depression cross‐sectional: *r* = −0.33, longitudinal: *r* = −0.29. Open PCC with mother and anxiety cross‐sectional: *r* = −0.04, longitudinal *r* = −0.17, father PCC and anxiety cross‐sectional: *r* = −0.09, longitudinal: *r* = −0.38.
						Longitudinal model: Direct path between open PCC with father at time 1 and depression (*β* = −0.19) and anxiety (*β* = −0.21) at time 2.
Depression & anxiety (CBCL^d^ anxious/depressed and withdrawn/depressed subscales)	Keim et al. (2017)^L^	USA	Cancer patients (125 52%), control group (60, 53%)	*M =* 13.5, *SD =* 2.4, range = 10–17	PACS	Longitudinal model: Higher mother openness in communication predicted lower withdrawn/depressed scores for children with advanced cancer *β* = 0.14. Fewer problems in communication with mothers at time 1 predicted lower withdrawn/depressed scores for children with advanced cancer at time 2, *β* = 0.14. Higher openness in communication with fathers at time 1 predicted lower anxious/depressed, *β* = 0.12, and withdrawn/depressed scores at time 2, *β* = 0.10.
General mental health (CBCL^d^)	Shin et al. (2010)^CS^	Korea	Adolescents from divorced families (178, 52.8%)	*M =* 13.0, *SD =* 0.4, range = 10–13	PACS	Correlation: PCC and general mental health: *r* = 0.42.
						Multiple regression: PCC did not predict mental health.
General mental health (SDQ^e^)	Wang et al. (2020)^CS^	China	Regular population school students (1969, 45.6%)	*M =* 13, range 11–17	PACS	Regression analyses: Mother open PCC and general mental health: *β* = −0.05 (*p* < 0.01), mother problem PCC and general mental health: *β* = 0.28 (*p* < 0.001). Father open PCC and general mental health: *β* = −0.01 (n.s.), father problem PCC and general mental health: *β* = 0.17 (*p* < 0.001).
General mental health (SDQ^e^)	Haukeland et al. (2020)^L^	Norway	Siblings of children with chronic disorders (99, 54.5%)	*M =* 11.5, *SD =* 2.0, range = 8–16	PCCS (McCarty et al.)	Correlations: PCC and general mental health at time 1: *r* = −0.18; time 2: *r* = −0.27; time 3: *r* = −0.20.
General mental health (SDQ^e^)	Haukeland et al. (2021)^I^	Norway	Siblings of children with rare disorders (56, 50%); control group (44, 50%)	Siblings of children with rare disorders (*M =* 11.3, *SD =* 1.7) and control group (*M =* 11.4, *SD =* 2.5)	PCCS (McCarty et al.)	Correlations: Sibling sample: Mother PCC and general mental health: *r* = −0.32. Father PCC and general mental health: *r* = −0.31.
						Control sample: Mother PCC and general mental health: *r* = −0.21. Father PCC and general mental health: *r* = −0.12. The at‐risk sample reported lower quality of PCC compared to controls (*d* = −0.93 for mothers and *d* = −0.61 for fathers).
General mental health (SDQ^e^)	Lu et al. (2020)^CS^	China	Left‐behind children of migrant fathers, living with mother (464, 45.7%)	Range = 11–17	PACS	Regression analyses: Mother open PCC and general mental health: *β* = −0.14 (*p* < 0.01), problem PCC and general mental health: *β* = −0.30 (*p* < 0.001). Father open PCC and general mental health: *β* = −0.04 (n.s.), problem PCC problem and general mental health: *β* = −0.15 (*p* < 0.05).
General mental health (SDQ^e^)	Wang et al. (2019)^CS^	China	Regular population school students (4565, 43.4%)	*M =* 13, *SD =* 1.3	PACS	Regression analyses: Mother open PCC and general mental health: *β* = −0.11, mother problem PCC and general mental health: *β* = 0.29. Father open PCC and general mental health: *β* = −0.04 (n.s.), father problem PCC and general mental health: *β* = 0.15.
General mental health (YSR^c^)	Wu and Chao (2011)^CS^	USA	Regular population high school students (634, 48.9%)	*M =* 16.0, *SD =* 0.6, range = 14–18	PACS	Discrepancies in parent‐adolescent open communication had no significant impact on internalizing nor externalizing mental health.
General mental health (YSR^c^)	Manczak et al. (2018)^L^	USA	Adolescent girls seeking psychiatric services (194, 100%)	*M =* 14.5, *SD =* 1.2, range 12–16	PACS	Correlations: Higher quality of mother‐child communication correlated with less internalizing (*r* = −0.35) and externalizing (*r* = −0.50) mental health difficulties in children.
						Multiple regression: The association between maternal depressive symptoms and internalizing (*β* = −0.01, *p* = 0.032) and externalizing (*β* = −0.11, *p* = 0.009) mental health difficulties was moderated by the quality of mother‐child communication so that higher quality communication predicted a weaker association.
General mental health (YSR^c^)	Kim and Park (2011)^CS^	USA	Regular population Korean immigrants (77, 39%)	*M =* 12.9, *SD =* 1.1, range = 11–15	PACS	Correlations: Mother PCC and internalizing: *r* = −0.26; externalizing: *r* = ‐0.37 father PCC and both internalizing and externalizing: *r =* −0.41.
						Multiple regression: Higher open father PCC levels were associated with fewer internalizing symptoms (*β* = −0.42, *p* < 0.001). The interaction term (father–PCC x enculturation gap) was a significant predictor of youths' internalizing symptoms (*β* = −0.23, *p* < 0.05).
General mental health – Externalizing problems (YSR^c^, subscale).	Park and Kim (2012)^CS^	USA	Regular population Korean immigrants (166, 45.8%)	*M =* 13.0, *SD =* 1.2, range 11–15	PACS	Correlations: Mother PCC and externalizing: *r* = −0.28, father PCC and externalizing: *r* = 0.31.
Psychosis (Kiddie Schedule for Affective disorders and Schizophrenie, present and Lifetime version K‐SADS‐PL, positive and negative syndrome scale)	Otero et al. (2011)^L^	Spain	Patients with first episode psychosis (100), control group (98)	Patients: *M =* 15.5, *SD =* 1.8; controls: *M =* 15.2; *SD =* 1.9, range = 9–17	PACS	ANCOVA: Group differences in PCC problems had an effect size of *d* = 0.64 for PCC with mothers and of *d* = 0.63 for fathers and remained significant after controlling for socioeconomic status (*p* < 0.001).
PTSD^f^ (child PTSD symptom scale)	Acuña and Kataoka (2017)^CS^	USA	Secondary students from four out of five high poverty schools (98, 44,9%)	*M =* 13.1, *SD* = 1.2	PACS	Correlations: Open PCC: *r* = −0.28 (total PTSD symptoms), *r* = −0.33 (avoidance PTSD symptoms), *r* = −0.26 (arousal PTSD symptoms), *r* = −0.11 re‐experiencing PTSD symptoms. Problematic PCC: *r* = 0.28 to 0.42 across all domains of PTSD symptoms.
						Multiple regression: Problem, but not open PCC was associated with more total PTSD symptoms (*β* = 0.42, *p* < 0.001) and avoidance symptoms (*β* = 0.20, *p* < 0.001).
PTSD^f^ (PTSD checklist of the diagnostic and statistical Manual of mental disorders)	Zhen et al., 2022 ^CS^	China	Regular population school students (683, 44.1%	*M =* 16.1, *SD =* 0.6, range 15–18	PACS	Correlations: Open PCC and PTSD symptoms: *r* = −0.25, problem PCC and PTSD symptoms: *r* = 0.36.
PTSD^f^ (PTSD checklist of the diagnostic and statistical Manual of mental disorders)	Zhou et al. (2021)^CS^	China	Adolescent earthquake survivors (620, 60.2%)	*M =* 15.6, *SD =* 1.6, range = 12–19	PACS	Correlations: Open PCC and PTSD: *r* = −0.21, problem PCC and PTSD: *r* = 0.28.
						SEM: Only open PCC showed a direct relationship with PTSD symptoms in the final SEM model (*β* = −0.11, *p* < 0.01).
Suicidal ideation (suicidal ideation subscale of the suicidal risk scale)	Kwok and Shek (2010a)^CS^	China	Regular population school students (5557, 46.9%)	*M =* 13.9, *SD =* 1.5, range = 11–18	Chinese father‐adolescent communication scale (FACS) & mother‐adolescent communication scale (MACS)	Correlations: Mother PCC and suicidal ideation: *r* = −0.42; father PCC and suicidal ideation: *r* = −0.36.
						Multiple regression: Direct effects on suicidal ideation (father PCC: *β =* −0.05, *p* < 0.05; mother PCC: *β =* −0.10, *p* < 0.001) and hopelessness (father PCC: *β =* −0.12, *p* < 0.001; mother PCC: *β* = −0.17, *p* < 0.001).
Suicidal ideation (suicidal ideation subscale of the suicidal risk scale)	Kwok and Shek (2010b)^CS^	China	Same as above	Same as above	Same as above	Correlations: Mother PCC and suicidal ideation: *r* = −0.42; father PCC and suicidal ideation: *r* = −0.36.
						Multiple regression with suicidal ideation as the dependent variable, standardized predictors were father PCC (*β* = −0.04, *p* < 0.05) and mother PCC (*β* = −0.13, *p* < 0.001), and interactions hopelessness × father PCC (*β* = −0.15, n.s.) and hopelessness × mother PCC (*β* = −0.21, *p* < 0.01).
Suicidal ideation (suicidal ideation subscale of the suicidal risk scale)	Kwok and Shek (2011)^CS^	China	Same as above	Same as above	Same as above	Correlations: Father PCC and suicidal ideation: *r* = −0.39 (females), *r* = −0.33 (males). Mother PCC and suicidal ideation: *r* = −0.43 (females), *r* = −0.42 (males).
						Multiple regression: Higher father PCC (*β* = −0.13, *p* < 0.001) and mother PCC (*β* = −0.23, *p* < 0.001) were associated with less suicidal ideation after controlling for demographic variables (parents' education, family income).

Abbreviations: CS, Cross‐sectional study; I, Interventional study; L, Longitudinal study.

^a^ Screen for Child Anxiety Related Disorders.

^b^ Center for Epidemological Studies Depression Scale.

^c^ Youth Self Report.

^d^ Child Behavior Checklist.

^e^ Strenghts and Difficulties Questionnaire.

^f^ Post‐traumatic stress disorder.

### Overview of studies and measures

Of all included papers, 25 were cross‐sectional studies, 11 were longitudinal studies, and one was an interventional study. The results of five studies were published in more than one of the papers included here: The papers Kwok and Shek (2010a, 2010b, 2011) all refer to the same cross‐sectional suicidal ideation study; Cai et al. (2021), Liu et al. (2019), Lu et al. (2020), Wang et al. (2019, 2020) all refer to the same cross‐sectional study of left‐behind children of parent migrant workers in rural China; Park & Kim (2011) and Kim & Park (2012) refer to the same cross‐sectional acculturation study; and five reports are part of the same longitudinal study (Finan et al., 2018; Ohannessian, 2012; Ohannessian & Vannucci, 2020; Pantaleao & Ohannessian, 2019; Simpson et al., 2020). The papers Haukeland et al. (2021) and (2022) refer to partly overlapping samples.

The Parent‐Adolescent Communication Scale (PACS; Barnes & Olson, 1985) was used in all but five studies. It contains openness in communication and communication problems as subscales. The remaining studies used McCarty's PCC Scale (PCCS; McCarty et al., 2003), Loeber's PCC Scale (PCCS; Loeber et al., 1998), the PCC Questionnaire (PCCQ; Yang & Zou, 2008), or the Father‐Adolescent/Mother‐Adolescent Communication Scale (FACS/MACS; Shek et al., 2006). Fourteen different standardized mental health measures were used. To sum up, we included 37 reports representing 26 studies, of which 86% used the PACS as the communication measure in relation to 14 various mental health measures.

### Quality assessment

All studies qualified for a clear research question that the collected data could inform. All reports were categorized as *Quantitative non‐randomized*. Two reports did not qualify for “yes” for the representativeness of the sample. All reports used standardized measures for adolescent mental health and PCC. In terms of completeness of outcome data, 12 reports qualified for “yes”, four for “no”, and 21 for “can't tell”. Nine reports did not qualify for “yes” regarding whether confounders like age, sex or socioeconomic status were accounted for in the design and analysis. The last category assessed whether the intervention was administered (or exposure occurred) as intended. This was applicable only to the intervention study (“yes”). To sum up, clarity of the research question and the use of standardized measures were methodological strengths that applied to all studies. The main methodological limitation that was evident for 24% of the studies was the lack of confounder control.

### General mental health

Four studies assessed PCC related to general adolescent mental health in community samples. Maternal open PCC was weakly linked to mental health, and paternal open PCC was not linked to mental health (Lu et al., 2020; Wang et al., 2019, 2020). In a sample of adolescents in divorced families, the correlation between PCC and mental health was medium (Shin et al., 2010). Another report found small and medium correlations for externalizing and internalizing behavior, respectively (Kim & Park, 2011, similar results from the same study in Park & Kim, 2012).

In an at‐risk sample of siblings of children with a chronic disorder (Haukeland et al., 2020, 2021), the at‐risk sample reported lower quality of PCC compared to controls with a large effect size for mother PCC and medium effects for father PCC. The correlation of child‐rated mental health with mother and father PCC was medium in this sample. However, mother‐rated mental health was not significantly related to PCC. In a clinical sample of adolescent girls referred to psychiatric services, the association between mother‐daughter communication and externalizing and internalizing behavior was large and medium, respectively. Furthermore, the quality of mother‐daughter communication fully buffered the impact of mothers' depressive symptoms on adolescents' internalizing and externalizing behavior (Manczak et al., 2018). To sum up, there were small to medium correlations between PCC quality and general mental health in community samples and medium to large in most at‐risk/clinical samples.

### Depression

Nine studies reported cross‐sectional data on the association between PCC and adolescent depression in community samples (see Table 1). The reported associations ranged from small to large. Most reports found medium correlations for total PCC (Chen et al., 2019; Ohannessian & Vannucci, 2020 (similar findings in Finan et al., 2018, Ohannessian, 2012; Pantaleao & Ohannessian, 2019, are based on overlapping samples of the same study); Schuster et al., 2013, Wang et al., 2022), as well as for open PCC (Ioffe et al., 2020; Rodrigues et al., 2020; Van Dijk et al., 2014). Similar results were obtained in two cross‐sectional at‐risk samples (Fernandez et al., 2022; Ying et al., 2018). Using latent class analysis, Ohannessian & Vannucci, 2020 also found that adolescents in a community sample with higher depressive symptoms were more likely to rate mother PCC as poor and father PCC as good. This indicates a higher association between mother PCC and adolescent depressive symptoms.

Papers from four longitudinal studies reported outcomes for community samples. Ohannessian and Vannucci (2020) found that depressive symptoms in adolescents predicted decreased PCC with mothers, but not fathers, 2 years later. Van Dijk et al. (2014) found small to medium associations between PCC and depression across four time points, each 1 year apart. In Ioffe et al. (2020), depressive symptoms 5 months later had medium associations with mother and father open PCC. However, only father open PCC predicted depressive symptoms in the final path model. Fite et al. (2014) reported that baseline PCC had a small link to depression 3 years later. One study was conducted on a clinical sample, reporting that better communication at baseline predicted lower depressive symptoms 1 year later in adolescents with advanced cancer but not in adolescents with non‐advanced cancer or controls (Keim et al., 2017). To sum up, the PCC and depression link ranges from small to large across various community samples, with most studies finding small to medium associations, some also over time. There was only one clinical study examining the association between PCC and depression.

### Anxiety

Five studies investigated PCC and adolescent anxiety in community samples. Simpson et al. (2020; similar findings in Ohannessian, 2012; Pantaleao & Ohannessian, 2019, based on overlapping samples) found small cross‐sectional associations between father PCC and both girls' and boys' anxiety. Boys' anxiety symptoms had small to medium associations with mother PCC. Fite et al. (2014) reported a small cross‐sectional association at age 16 years and also 3 years later. Similar results were found by Van Dijk et al. (2014), who observed small associations between open PCC and anxiety across four time points, each 1 year apart. Ioffe et al. (2020) found very small cross‐sectional correlations between open PCC and anxiety for mothers and fathers. Anxiety symptoms 5 months later had small and medium associations with maternal and paternal open PCC, respectively. In the final path model, only paternal open PCC was retained, indicating that communication quality with the father is a predictor for adolescent anxiety. In other multiple regression models, open PCC did not predict adolescent anxiety (Varela et al., 2009). To sum up, the PCC and anxiety links were mainly small, and all rested on cross‐sectional data from community samples.

### Psychosis

The association between PCC and psychotic symptoms was examined in only one study (Otero et al., 2011). Group comparisons showed that healthy controls reported better PCC than adolescents with a first psychotic episode, with a medium effect for adolescent‐rated mother‐child and father‐child communication. In the clinical sample, more problems in PCC at baseline were associated with a higher degree of psychopathology and a lower clinical improvement 1 year later.

### Suicidal ideation

Two studies investigated the relationship between PCC and suicidal ideation. Kwok et al. (2010a, for results from the same sample, see Kwok et al., 2010b, 2011) found a medium association between father and mother PCC and suicidal ideation. In a general regression model, mother PCC demonstrated a standardized small effect compared to father PCC, with mother PCC and adolescent hopelessness as a significant interaction term. In another study, Lu et al. (2020) found that mother, not father, PCC reduced the odds of suicidal ideation in migrant families.

### Post‐traumatic stress disorder symptoms

Three studies examined PCC and Post‐traumatic stress disorder (PTSD) symptoms. Zhen et al. (2022) reported medium associations between symptoms of posttraumatic stress disorder (PTSD) and open and problem PCC. In their general population study on the effects of the COVID‐19 pandemic, the effects of open and problem PCC on PTSD were partly mediated by self‐compassion and self‐disclosure. Acuña and Kataoka (2017) investigated the association in a sample of school students referred to counseling and found medium to large associations. Problem PCC, but not open PCC, predicted PTSD in the final model. In a study with adolescent earthquake survivors, Zhou et al. (2021) found medium associations between PTSD and open and problem PCC, respectively. In their final model, only open PCC was directly related to PTSD; no indirect paths were identified.

### Addictive internet use/gaming

Three papers addressed the association between addictive internet use or gaming and PCC in community samples. Cai et al. (2021) found that the quality of mother‐ and father‐child communication had a small protective effect against internet addiction. Kim et al. (2018) found that the risk of internet gaming disorders in middle school students was medium correlated with father‐child, but not with mother‐child communication. Liu et al. (2019) also reported medium correlations between high school students' PCC and pathological internet use.

### Addictive behavior: Substance use

Five of the included studies addressed the association of substance use and PCC in addition to other outcomes. Bireda and Pillay (2018), Chen et al. (2019), Ohannessian (2012), Ohannessian and Vannucci (2020), and Wang et al. (2020) reported associations between PCC and substance‐related addictive behavior. None of the studies used a standardized questionnaire to assess addictive behavior. Therefore, results are not included in this review according to eligibility criteria.

## DISCUSSION

We systematically reviewed 37 papers, representing 26 studies, examining the association between adolescent‐rated PCC quality and adolescent mental health. Our findings indicated that the quality of PCC and adolescent mental health are negatively associated, with small to medium effects between PCC and different mental health constructs. These findings are in line with theoretical proposals based on attachment theory and interpersonal psychotherapy (Luyten et al., 2020; O’Shea et al., 2014). This is also in line with the conceptual assumption that PCC is closely associated with constructs such as parent‐child attachment and/or the parent‐child relationship (Feddern Donbaek & Elklit, 2014; Heyman, 2001). The overall PCC‐mental health association should be considered in light of several nuances that were identified across the studies. Importantly, the strength of the PCC‐mental health association depends on parent and adolescent sex and gender interactions in the parent‐adolescent dyads. Across studies, the findings were inconsistent, which might be related to different (sub‐)cultural contexts of the included studies and different roles of fathers and mothers (Updegraff et al., 2002).

Considering our findings in light of existing models proposed for the PCC‐mental health association is also important. Overall, the evidence for various models was mixed. The reciprocal process model suggests that mental health and PCC quality mutually influence each other (Prescott & Le Poire, 2002). Some, but not all, of the findings from the longitudinal studies in this review were consistent with this model assumption. Differences in age (adolescents) and gender (parents and adolescents) seem to play a role. Mother PCC appeared as a stronger predictor for older adolescents, and father PCC appeared as a stronger predictor for younger adolescents. Further, mother PCC appeared as a stronger predictor for depressive symptoms in girls, and father PCC appeared as a stronger predictor for anxiety and depressive symptoms in boys.

In the risk/protective factor model, PCC is considered a risk or protective factor in relation to adolescent mental health (Segrin, 2006). Again, the evidence from longitudinal studies was mixed and does currently not fully support this model. Partly in line with this assumption, one longitudinal study presented evidence that PCC quality moderated the association between maternal depressive symptoms and mental health in adolescent girls (Manczak et al., 2018). As in the case of the reciprocal process model, gender and age interacted in moderating outcomes. This is in line with previous research (Colarossi & Eccles, 2003; Meadows et al., 2006).

Finally, in the mediation model, PCC is considered a mediator that explains the association between risk factors and adolescent mental health (Riesch et al., 2006). None of the studies in the current review were designed as a mediation study in the strict sense (i.e., where PCC was considered as a potential mediator for the relation between two other constructs and timewise measured between the other constructs). To sum up, the current evidence base is not sufficient to fully buffer existing models. However, the studies were not necessarily designed to test models, and various factors, like the time periods between measurements, were between 5 months and several years, so many other factors may have influenced the association between PCC and adolescent mental health and the role of risk factors in the meantime. Theoretically based variable inclusion and timing of assessments is warranted in the PCC research field.

We identified studies in several domains of adolescent mental health. To an extent, the strength of the PCC‐mental health association varied across mental health domains. Concerning depression, medium associations between PCC and adolescent depression were typically reported, and the results from longitudinal studies underscored this relationship. This is consistent with theoretically based expectations, such as Goodman and Gotlib's (1999) model of mechanisms of disorder transmission. In the studies that investigated both anxiety and depression, results on anxiety showed smaller associations with PCC compared to depression. A theoretical explanation for this finding might be that one evolutionary function of showing anxiety is to activate care and support in the social environment, and a relatively improved PCC may be the result of this activation process, whereas depressive behavior may lead to increased conflict with the social environment and result in a negative feedback loop (Lebowitz et al., 2014; Wittenborn et al., 2016). In contrast to anxiety, the association between PCC and psychosis was medium and in line with theoretical expectations, assuming parental communication deviance and expressed emotions to be a major factor in the course of the disease (de Sousa et al., 2014). Even though similar results were found in community and at‐risk samples, there is not yet enough information on differential pathways or developmental conditions that moderate or mediate the relationship.

Notably, we found only one study on PCC and psychosis and no studies on PCC and somatoform, obsessive‐compulsive, or neurodevelopmental disorders. Regarding substance‐related addictive behavior, no studies using standardized measures for substance abuse were found. In terms of study design, only one study was found to investigate the relationship based on an interventional design, indicating that interventions focusing on PCC can improve child mental health in an at‐risk group (Haukeland et al., 2021).

### Limitations

To our knowledge, this review is the first to give a comprehensive overview of the relationship between PCC quality and adolescent mental health. A major limitation of the review is the exclusive focus on adolescent self‐reported PCC questionnaires, as studies based on parent‐rated PCC measures alone were not eligible for inclusion. Depending on the mental health area, parent‐ or observer‐rated PCC outcomes may differ widely from adolescent‐rated ones (Hadley et al., 2013). Another limitation is that although the comparability of the results reported in this review is high since most studies used the same PCC questionnaire (PACS), the psychometric quality, even of widely used instruments, is still under discussion (Zapf et al., 2023). Furthermore, PCC also overlaps with constructs such as attachment or parent‐adolescent relationship that were not included in this review. A more comprehensive perspective would have to include overlapping constructs as well. Linked to this problem, another limitation is that until now, too little has been done to theoretically define and operationalize PCC, leaving only a “shaky foundation” for research and clinical practice (Heyman, 2001). Therefore, even if the results of this review show some consistency, it is hard to tell what instruments like the PACS measure precisely. It is also important to note that the evidence provided in the current review does not permit conclusions about causal relationships. It remains unclear whether PCC quality acts as a resilience/risk factor or is co‐created by adolescent mental health.

### Implications and conclusion

This review has implications for adolescent mental health practitioners and researchers. In terms of clinical practice, PCC quality is likely to both affect and be affected by adolescent mental health. Across the included studies, PCC quality, as perceived by the adolescent, was linked to adolescent mental health with mostly small to medium associations. Given the associations were not larger, on the one hand, our findings do not build a very strong case for saying that PCC should be assessed or targeted to influence adolescent mental health. However, given the vast number of variables that are likely to influence adolescent mental health, identifying some factors consistently associated with it, albeit small to medium, does provide some direction for the field. Our findings suggest that PCC may be considered in mental health practice, both in terms of assessing its quality and potentially by tailoring interventions to enhance PCC. These may be some of several means to promote adolescent mental health. Note that in the domains of general mental health and depression, these associations were stronger than in domains like internet‐related addictive behavior. Further, in some domains like suicidal ideation, parental roles had relevant effects on the strength of the association. Especially in cases of depression, PTSD, and psychosis, a focus on PCC quality may have therapeutic relevance.

Regarding implications for research, our findings indicate that PCC is a relevant variable to consider in assessing mental health over time. Future studies should also consider PCC beyond the perspective of youth self‐report. As only one intervention study has been conducted yet, further research with interventional designs would broaden our understanding of underlying mechanisms. In addition, research on yet unstudied areas like neurodevelopmental, somatoform, or obsessive‐compulsive disorders or research on socio‐economic subgroups and their specific support needs in terms of PCC would enrich clinical practice. Regarding the existing models, research on the reciprocal process model should entail shorter periods between measurements and time series models. In the research on the risk/protective factor model, existing studies should be replicated to better understand the role of the interaction of age and gender. Research on the mediation model would benefit from more longitudinal research and research on both mothers and fathers.

We conclude that PCC is moderately associated with several mental health domains, can be considered in clinical practice, and that further research is needed to clarify the role of PCC from other perspectives than adolescent self‐report and for moderators and mediators of the PCC and mental health associations.

## AUTHOR CONTRIBUTIONS

**Holger Zapf**: Conceptualization; Formal analysis; Investigation; Methodology; Project administration; Writing – original draft; Writing – review & editing. **Johannes Boettcher**: Data curation; Formal analysis; Investigation; Validation; Writing – review & editing. **Yngvild Haukeland**: Data curation; Formal analysis; Investigation; Validation; Writing – review & editing. **Stian Orm**: Data curation; Formal analysis; Investigation; Software; Validation; Writing – review & editing. **Sarah Coslar**: Formal analysis; Investigation; Resources; Writing – review & editing. **Krister Westlye Fjermestad**: Methodology; Supervision; Validation; Writing – review & editing.

## CONFLICT OF INTEREST STATEMENT

The authors have declared that they have no competing or potential conflicts of interest.

## ETHICAL CONSIDERATIONS

No ethical approval was required for this research review.

## Data Availability

The data that support the findings of this study are available on request from the corresponding author.
